# Within-Subject Interlaboratory Variability of QuantiFERON-TB Gold In-Tube Tests

**DOI:** 10.1371/journal.pone.0043790

**Published:** 2012-09-06

**Authors:** William C. Whitworth, Lanette R. Hamilton, Donald J. Goodwin, Carlos Barrera, Kevin B. West, Laura Racster, Laura J. Daniels, Stella O. Chuke, Brandon H. Campbell, Jamaria Bohanon, Atheer T. Jaffar, Wanzer Drane, David Maserang, Gerald H. Mazurek

**Affiliations:** 1 Division of Tuberculosis Elimination, Centers for Disease Control and Prevention, Atlanta, Georgia, United States of America; 2 Department of Medicine, Tripler Army Medical Center, Honolulu, Hawaii, United States of America; 3 Epidemiology Services Branch, United States Air Force School of Aerospace Medicine, Brooks City-Base, Texas, United States of America; 4 Department of Occupational Medicine/TB Prevention/Deployment Medicine, Wilford Hall Medical Center, Reid Clinic, Lackland Air Force Base, Texas, United States of America; 5 CDC Foundation, Atlanta, Georgia, United States of America; 6 Northrop Grumman Information Systems Sector, Atlanta, Georgia, United States of America; 7 Distinguished Professor Emeritus of Biostatistics, University of South Carolina, Columbia, South Carolina, United States of America; 8 Applied Technology Center, United States Air Force School of Aerospace Medicine, Brooks City-Base, Texas, United States of America; McGill University, Canada

## Abstract

**Background:**

The QuantiFERON®-TB Gold In-Tube test (QFT-GIT) is a viable alternative to the tuberculin skin test (TST) for detecting *Mycobacterium tuberculosis* infection. However, within-subject variability may limit test utility. To assess variability, we compared results from the same subjects when QFT-GIT enzyme-linked immunosorbent assays (ELISAs) were performed in different laboratories.

**Methods:**

Subjects were recruited at two sites and blood was tested in three labs. Two labs used the same type of automated ELISA workstation, 8-point calibration curves, and electronic data transfer. The third lab used a different automated ELISA workstation, 4-point calibration curves, and manual data entry. Variability was assessed by interpretation agreement and comparison of interferon-γ (IFN-γ) measurements. Data for subjects with discordant interpretations or discrepancies in TB Response >0.05 IU/mL were verified or corrected, and variability was reassessed using a reconciled dataset.

**Results:**

Ninety-seven subjects had results from three labs. Eleven (11.3%) had discordant interpretations and 72 (74.2%) had discrepancies >0.05 IU/mL using unreconciled results. After correction of manual data entry errors for 9 subjects, and exclusion of 6 subjects due to methodological errors, 7 (7.7%) subjects were discordant. Of these, 6 (85.7%) had all TB Responses within 0.25 IU/mL of the manufacturer's recommended cutoff. Non-uniform error of measurement was observed, with greater variation in higher IFN-γ measurements. Within-subject standard deviation for TB Response was as high as 0.16 IU/mL, and limits of agreement ranged from −0.46 to 0.43 IU/mL for subjects with mean TB Response within 0.25 IU/mL of the cutoff.

**Conclusion:**

Greater interlaboratory variability was associated with manual data entry and higher IFN-γ measurements. Manual data entry should be avoided. Because variability in measuring TB Response may affect interpretation, especially near the cutoff, consideration should be given to developing a range of values near the cutoff to be interpreted as “borderline,” rather than negative or positive.

## Introduction

Interferon gamma (IFN-γ) release assays (IGRAs) are designed to detect both latent *Mycobacterium tuberculosis* infection (LTBI) and infections manifesting as active tuberculosis disease, collectively referred to as *M. tuberculosis* infection (MtbI). IGRAs are a popular, viable, and often preferred alternative to the traditional tuberculin skin test (TST) in some settings [1]–[3]. Despite inadequacies in diagnostic standards for identifying MtbI, numerous studies have assessed the sensitivity and specificity of IGRAs [2]–[4]. However, few studies have assessed the within-subject variability of IGRA results. Within-subject variability includes differences in test results due to both subject fluctuations and test performance fluctuations. Excessive variability in IGRA results may limit their utility for detecting MtbI. A limited number of studies have assessed IGRA variability among people where treatment might affect serial test results [5]–[9] or among contacts, healthcare workers (HCW), or residents of high-TB burden countries where ongoing transmission may affect serial IGRA results [10]–[24]. Rarely have investigators examined variability due solely to test performance fluctuations on blood collected at the same time [13], [20]. No published investigation has addressed variability when IGRAs are performed in different laboratories on blood collected at the same time.

The QuantiFERON®-TB Gold In-Tube test (QFT-GIT, Cellestis Limited, Carnegie, Victoria, Australia) is one of two commercially available IGRAs currently in use in the U.S. The goal of this study was to determine the within-subject variability of the QFT-GIT when performed in different laboratories on blood collected at the same time and to investigate potential reasons for variability.

## Methods

### Ethics Statement

The Centers for Disease Control and Prevention (CDC) and Wilford Hall Medical Center human subjects institutional review boards approved this study. All subjects provided written informed consent.

### Subject Selection

Subjects were recruited from among Air Force and CDC staff located in San Antonio, Texas, and Atlanta, Georgia, respectively, as part of a larger study investigating parameters that affect QFT-GIT variability. Prior unpublished assessments among a similar cohort found a broad range of IFN-γ measurements, and that 40% to 50% of persons with self-reported prior positive TST results were positive by QFT-GIT as compared to <3% for the general U.S. population. To increase the proportion of subjects with positive QFT-GIT results and to assess subjects with a continuous range of IFN-γ measurements, including those with IFN-γ measurements near the cutoff separating positive and negative interpretations, only persons with self-reported prior positive TST results were recruited. Exclusion criteria were age of less than 18 years or a history of an adverse reaction to TST (e.g., blistering, scarring, or anaphylaxis). All subjects completed a detailed study questionnaire.

### QFT-GIT Procedure

Blood from each subject was collected at a single sitting into three sets of QFT-GIT tubes so that the assay could be completed in three different labs (Lab1, Lab2, and Lab3), all with extensive experience and demonstrated proficiency. Approximately 1 mL of blood was collected into three tubes containing only heparin (Nil tube); three tubes containing heparin, dextrose, and the mitogen phytohemagglutinin A (Mitogen tube); and three tubes containing heparin, dextrose, and Mtb antigens (TB Antigen tube). Mtb antigens consisted of a single mixture of peptides representing ESAT-6, CFP-10, and TB7.7 as described in the package insert. Tubes with identical lot numbers were used. Tube contents were mixed with a Stuart rock-and-roll mixer (SciTech Instruments, Inc., Franklin, NJ) for 3 minutes at 33 RPM with the tube cap end lowered 20° to ensure that the entire inner surface of each tube was covered with blood. Within 1 hour of collection, the tubes were placed upright in an incubator at 37+/−0.5°C. The tubes were incubated for 23 to 24 hours, after which they were centrifuged at 3,000 g for 10 minutes. Centrifuged tubes were stored and shipped at 2°C to 8°C. Temperatures during incubation, storage, and shipping were confirmed with a SL300 temperature data logger (SupCo, Allenwood, NJ). The IFN-γ concentrations in plasmas from the Nil tube, the TB Antigen tube, and the Mitogen tube (abbreviated Nil, TB, and Mitogen, respectively) were determined by enzyme-linked immunosorbent assay (ELISA), performed 13 to 15 days after blood collection using reagents included in QFT-GIT kits. No attempt was made to assure that QFT-GIT ELISA kits had identical lot numbers. All test parameters were within specifications stipulated in the QFT-GIT package insert. The TB Response was calculated by subtracting Nil from TB, and Mitogen Response was calculated by subtracting Nil from Mitogen.

Lab1 and Lab2 performed ELISAs with the aid of Triturus automated ELISA workstations (Grifols USA, Inc., Miami, FL) and used eight IFN-γ standard calibrators (8, 4, 2, 1, 0.5, 0.25, 0.125, and 0 IU/mL) in duplicate to create standard curves. In contrast, Lab3 performed ELISAs with the aid of a DSX automated ELISA workstation (Dynex Technologies, Chantilly, VA) and used four IFN-γ standard calibrators (4, 1, 0.25, and 0 IU/mL) in duplicate to create standard curves after local validation of the method. Raw optical density (OD) values were transferred electronically at Lab1 and Lab2 and manually entered at Lab3. Plasma IFN-γ concentrations were determined using software developed by Cellestis (QuantiFERON®-TB Gold In-Tube Analysis Software v2.17.2) and with a Microsoft Access 2007 v12 database (Microsoft, Inc., Seattle, WA), developed at the CDC. The CDC database differs from the software provided by Cellestis in that INF-γ concentrations were not truncated at 10 IU/mL or rounded prior to subtracting Nil to determine TB Response and Mitogen Response.

Test results were interpreted as indicated in the Cellestis package insert and CDC guidelines [2], [25]. The interpretation was “positive” if the Nil was ≤8.0 IU/mL and the TB Response was ≥0.35 IU/mL and ≥25% of the Nil. The interpretation was “negative” if the Nil was ≤8.0 IU/mL, the Mitogen Response was ≥0.5 IU/mL, and the TB Response was <0.35 IU/mL or <25% of the Nil. The interpretation was “indeterminate” if (a) the Nil was >8.0 IU/mL or (b) the Nil was ≤8.0 IU/mL, the Mitogen Response was <0.5 IU/mL, and the TB Response was <0.35 IU/mL or <25% of the Nil. For subjects with discordant interpretations, discrepancies in TB Response >0.05 IU/mL, or unusual IFN-γ measurements [26], results were recalculated based on verified OD values entered directly from the ELISA reader printout and used to create a reconciled dataset.

### Statistical Methods

For assessment of variability in test interpretations (variability in qualitative results), the percentage of subjects with concordant results from tests performed at the three different labs was determined. For each pair of labs, positive agreement, negative agreement, and agreement beyond chance (Cohen's kappa statistic) were calculated. For the assessment of variability in quantitative results, Nil, TB, and TB Response distributions were compared using the Wilcoxon signed-rank test. Five additional indices of quantitative variability, the last two of which were derived from the standard deviation of the differences (SD_diff_), were examined including (1) within-subject coefficient of variation (W-S CV%), (2) intraclass correlation coefficient (ICC), (3) mean difference between two labs (bias), (4) the smallest detectable difference (SDD), and (5) the within-subject standard deviation (W-S SD). SDD = 1.96*SD_diff_ and is the smallest change in a second measurement that must occur to detect a change above the variability (e.g., noise) with 95% confidence [27], [28]. W-S SD = ±(SD_diff_/√2) [29] and represents 68% of the variation expected around the true value [30]. Limits of agreement (LOA) = bias ± SDD and encompass the range around the bias that contains 95% of within-subject differences [31]. ICCs were calculated using the SAS macro ICC_SAS [32]. W-S CV% was calculated as described by Bland (root mean square approach) [33] for Nil and TB but estimated for TB Response using the formula √[(W-S CV%_TB_)^2^+(W-S CV%_Nil_)^2^]. The W-S CV%s for the TB Response could not be directly determined due to inflation caused by zeroes and low means in the denominator (a result of subjects with both positive and negative TB Response values). A confidence level of 0.95 was used in all hypothesis tests. Stratified analyses for quantitative indices were performed on concordant positive, concordant negative, and discordant groups and three groups stratified by mean TB Response of <0.10 IU/mL, 0.10 through 0.60 IU/mL, and >0.60 IU/mL. Indices of variability were not reported for groups with less than 10 subjects to avoid inaccuracies due to small sample size. SAS v9.2 (SAS, Cary, NC) and “Analyse-It” v2.22 for Excel (Analyse-It Software, Ltd., Leeds, UK) were used to perform the analyses.

## Results

### Subject Characteristics

**Figure 1:**
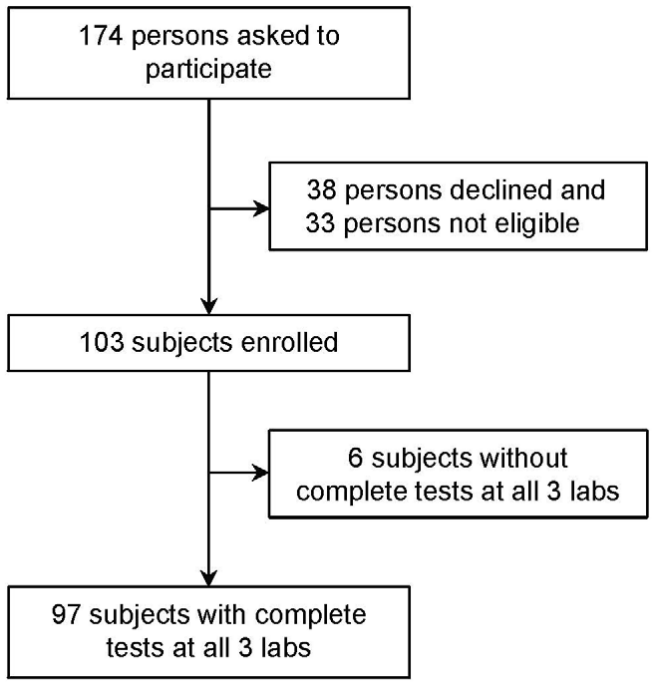
Study participation diagram.

Study participation is depicted in Figure 1. Of the 174 people asked to participate, 103 consented, and 97 had QFT-GIT tests completed in all three labs. Characteristics of study subjects are shown in Table 1.

**Table 1:** Subject characteristics.

Characteristic	Category	n (%)
Age, years	20–29	8 (8.2%)
	30–39	23 (23.7%)
	40–49	29 (29.9%)
	50–59	28 (28.9%)
	≥60	9 (9.3%)
Gender	M	47 (48.5%)
	F	50 (51.5%)
Race/Ethnicity	White, non-Hispanic	46 (47.4%)
	Black, non-Hispanic	19 (19.6%)
	Hispanic	15 (15.5%)
	Asian/Pacific	13 (13.4%)
	Native American	0 (0.0%)
	Other	4 (4.1%)
Year of Last Positive TST	1950–1959	1 (1.0%)
	1960–1969	4 (4.1%)
	1970–1979	5 (5.2%)
	1980–1989	12 (12.4%)
	1990–1999	38 (39.2%)
	2000–2009	37 (38.1%)
Received Therapy for TB	Yes	2 (2.1%)
	No	95 (97.9%)
Received Therapy for LTBI	Yes	78 (80.4%)
	No	19 (19.6%)
Known Exposure to Active TB	Yes	36 (37.1%)
	No/Unknown	61 (62.9%)
Received BCG Vaccine	Yes	22 (22.7%)
	No/Unknown	75 (77.3%)
Region of Birth	United States and Canada	66 (68.0%)
	Central America/Caribbean	9 (9.3%)
	Asia	7 (7.2%)
	Southeast Asia	4 (4.1%)
	Pacific	3 (3.1%)
	Europe/Russia	3 (3.1%)
	Africa	2 (2.1%)
	Middle East	2 (2.1%)
	South America	1 (1.0%)
Years Lived Outside USA	None	36 (37.1%)
	1–10	40 (41.2%)
	11–20	12 (12.4%)
	21–30	8 (8.2%)
	31–40	1 (1.0%)

### Qualitative Results Using Original Data

Comparisons of test interpretations among all three labs using original (unreconciled) data are shown in Table 2. No QFT-GIT result was indeterminate. Eleven of 97 subjects (11.3%) had discordant results. Comparisons of test interpretations between pairs of labs are shown in Table 3. Discordance ranged from 5.2% to 10.4% using original data. Nil concentrations, TB Responses, and QFT-GIT interpretations are shown in Table S1 for the 11 subjects with discordant interpretations using original data. Of these 11 subjects, 4 (36.4%) had all TB Responses within 0.25 IU/mL of the 0.35 IU/mL cutoff.

**Table 2:** Summary comparisons of QuantiFERON-TB Gold In-Tube test interpretations performed on the same subjects in three labs.

Results compared	n	All 3 positive	All 3 negative	2 positive & 1 negative	1 positive & 2 negative	Total discordant (%)
Lab1, Lab2, & Lab3 (original data)	97	39	47	6	5	11 (11.3%)
Lab1, Lab2, & Lab3 (reconciled data)	91	35	49	4	3	7 (7.7%)

**Table 3:** Pairwise comparisons of QuantiFERON-TB Gold In-Tube test interpretations performed on the same subjects in three labs.

					% Agreement			% Discordant	
Results compared (Group 1 vs. Group 2)	Both positive	Both negative	Positive*/negative	Negative*/positive	Overall	Positive	Negative	Overall	Kappa
Lab1 vs. Lab2 (original data)	42	50	3	2	94.8	89.4	90.9	5.2	0.90
Lab1 vs. Lab3 (original data)	40	47	5	5	89.7	80.0	82.5	10.4	0.79
Lab2 vs. Lab3 (original data)	41	49	3	4	92.8	85.4	87.5	7.2	0.85
Lab1 vs. Lab2 (reconciled data)	36	50	3	2	94.5	87.8	90.9	5.5	0.89
Lab1 vs. Lab3 (reconciled data)	36	49	3	3	93.4	85.7	89.1	6.6	0.87
Lab2 vs. Lab3 (reconciled data)	37	51	1	2	96.7	92.5	94.4	3.3	0.93

* = Group1/Group2.

### Quantitative Results Using Original Data

Median and mean Nil, TB, and TB Response values using original data are shown in Table 4. Seventy-two (74.2%) subjects had discrepancies in TB Response >0.05 IU/mL. One subject had all three Nil values >0.7 IU/mL and three other subjects had at least one NIL value >0.4 IU/mL. No subjects had TB Responses <−0.35 IU/mL or Mitogen Responses <−0.5 IU/mL. Indices of quantitative variability in original Nil, TB, and TB Response are shown in Table S2.

**Table 4:** Median and mean IFN-γ measurements for QuantiFERON-TB Gold In-Tube tests performed on the same subjects in three labs.

Source	n	Nil		TB		TB Response	
		Median	Mean	Median	Mean	Median	Mean
Lab1 (original) unstratified	97	0.07	0.10	0.27	4.11	0.15	4.01
Lab2 (original) unstratified	97	0.06	0.09	0.24	3.88	0.19	3.79
Lab3 (original) unstratified	97	0.07	0.11	0.40	5.92	0.26	5.81
Lab1 (reconciled)							
Unstratified *	91	0.07	0.09	0.18	3.11	0.08	3.01
Concordant Positive†	35	0.07	0.11	3.93	7.85	3.31	7.74
Concordant Negative§	49	0.07	0.08	0.08	0.10	0.01	0.02
Lab2 (reconciled)							
Unstratified *	91	0.06	0.09	0.16	2.91	0.09	2.83
Concordant Positive†	35	0.07	0.11	4.47	7.37	4.23	7.26
Concordant Negative§	49	0.06	0.07	0.08	0.09	0.01	0.02
Lab3 (reconciled)							
Unstratified *	91	0.07	0.11	0.32	2.94	0.18	2.33
Concordant Positive†	35	0.07	0.12	3.72	6.04	3.25	5.91
Concordant Negative§	49	0.07	0.10	0.11	0.14	0.02	0.05

Nil = IFN-γ concentrations (IU/mL) in plasma from the Nil tube of the QuantiFERON-TB Gold In-Tube test (QFT-GIT); TB = IFN-γ concentrations (IU/mL) in plasma from the TB tube of QFT-GIT; TB Response = TB minus Nil.

* Includes 7 subjects with discordant QFT-GIT interpretations.

† Concordant positive among results from all 3 labs.

§ Concordant negative among results from all 3 labs.

### Recognition of Data Entry and Methodological Errors

No errors in electronically transferred data were identified. Two types of manual data entry errors at Lab3 were identified, affecting results for nine subjects. The first type of error was a misalignment of results for eight subjects so that TB, Nil, and TB Response values were assigned to the wrong subjects. The second type of error, affecting a ninth subject, occurred as a result of a misplaced decimal point due to human error that caused inaccuracy in reported TB and TB Response values. A line listing of QFT-GIT results from these nine subjects is shown in Table S3. These errors were corrected in the reconciled dataset. A third type of error was recognized for six subjects who had extremely high IFN-γ concentrations reported for TB values in Lab3 (range 37.4 to 102.5 IU/mL) when compared to Lab1 and Lab2 (range 8.6 to 18.4 IU/mL) and when compared to other Lab3 TB values (all >7 times the interquartile range of 3.33 IU/mL). TB and TB Response values for these six subjects and a seventh subject with the next highest Lab3 TB and TB Response values are shown in Table S4. The large discrepancies and high TB values reported by Lab3 were due to misinterpreted OD values reported by the ELISA workstation. OD values above the working range of the Lab3 reader were reported as “9.999”, resulting in calculation of exaggerated and inaccurate IFN-γ concentrations. This was a methodological error. OD values above the working range were reported in the other labs as “OWR” (outside of working range), thus preventing calculation of an IFN-γ concentration. Because the ODs reported as “9.999” could not be verified for the six subjects with exaggerated TB values, data from these six subjects were excluded from the reconciled dataset.

### Qualitative Results Using Reconciled Data

Comparisons of test interpretations among all three labs using reconciled data are shown in Table 2. No QFT-GIT result was indeterminate. Seven of 91 subjects (7.7%) had discordant results after data were reconciled. Comparisons of test interpretations between pairs of labs are shown in Table 3 using reconciled data. Nil concentrations, TB Responses, and QFT-GIT interpretations are shown in Table S1 for the 7 subjects with discordant interpretations using reconciled data. Of these seven, six (85.7%) had all TB Responses within 0.25 IU/mL of the 0.35 IU/mL cutoff. Of 12 subjects who had one or more TB Responses within 0.25 IU/mL of the cutoff, 7 (58.3%) had discordant QFT-GIT interpretations, while none of the 72 subjects with no TB Response in this range had discordance.

### Quantitative Results Using Reconciled Data

**Figure 2:**
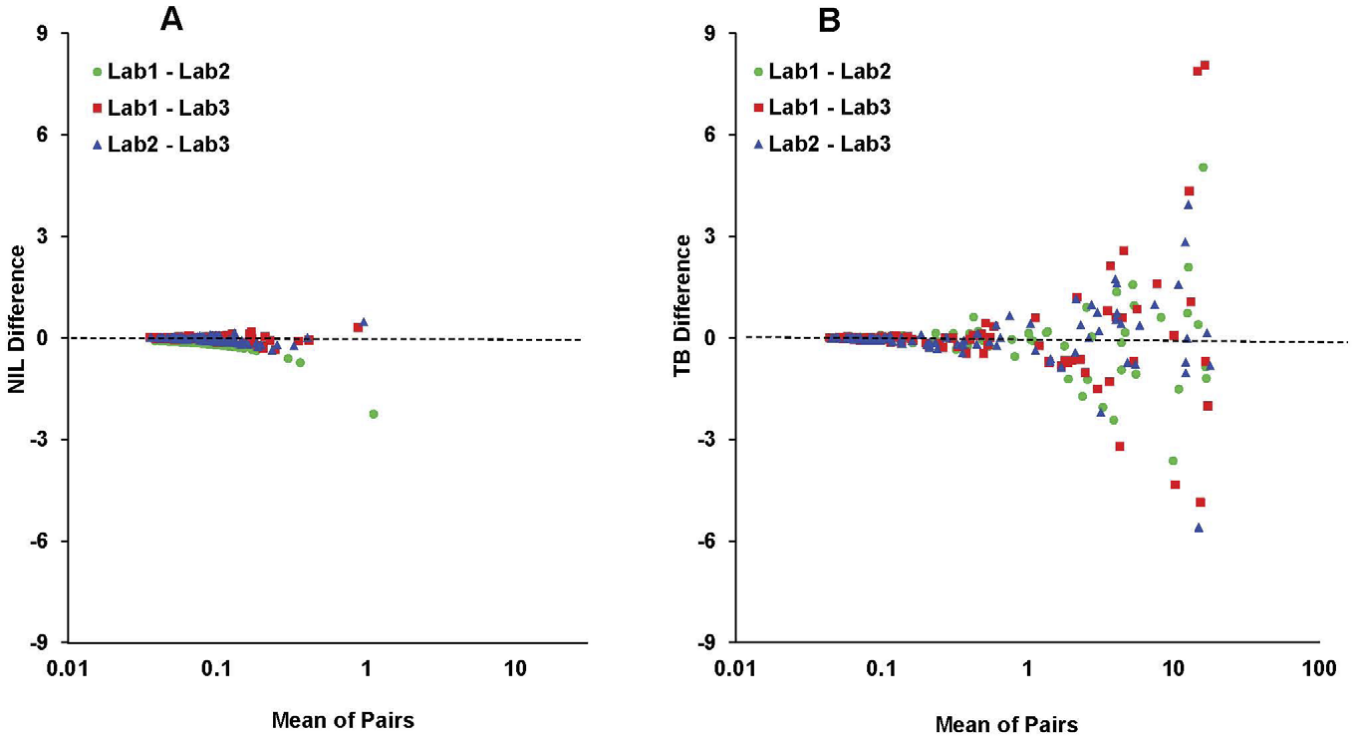
Difference (Bland-Altman) plots. Difference (Bland-Altman) plots for Nil (panel A) and TB (panel B). Differences (y-axis) and means of pairs (x-axis) are in IU/mL IFN-γ.

Median and mean Nil, TB, and TB Response values using reconciled data are shown in Table 4. NIL values >0.4 IU/mL did not change. No subjects had TB Responses <−0.35 IU/mL or Mitogen Responses <−0.5 IU/mL. Examination of the reconciled data with Bland-Altman difference plots (Figure 2) showed that variation increased as the mean of the paired measurements increased. For this reason, stratified analyses were performed. Among concordant negatives, TB Responses in Lab3 were significantly greater than in Lab1 (p<0.001, Wilcoxon signed-rank test) or Lab2 (p = 0.002). TB values followed a similar pattern (p = 0.01 and 0.001, respectively). Among concordant positives, TB and TB Responses in Lab2 were significantly greater than in Lab3 (p = 0.01 for both). No significant differences were seen for any of the Nil comparisons.

Indices of quantitative variability in reconciled Nil, TB, and TB Response values are shown in Table 5. Bias and LOA showed greater variability in TB Response among subjects with concordant positive interpretations than those with concordant negative interpretations. Bias in TB Response ranged from 0.00 IU/mL when data from Lab1 and Lab2 were compared for subjects with concordant negative interpretations to 1.82 IU/mL when data from Lab1 and Lab3 were compared for subjects with concordant positive interpretations. SDD ranged from 0.08 to 9.61 IU/mL in these groups, respectively. Indices for TB Response variability tracked indices of variability for TB. Nil values were less variable between strata and between labs than TB or TB Response values. W-S SD followed a similar trend with variability of concordant positives > variability of total population > variability of concordant negatives. Examination of ICC revealed that concordant negatives were less correlated than concordant positives. Variability adjusted for each subject's mean value (W-S CV%) was similar for subjects with concordant negative and concordant positive results for Lab1 vs. Lab2, but much larger in concordant negatives for TB and TB Response when Lab1 or Lab2 was compared to Lab3.

**Table 5:** Variability in Quantiferon-TB Gold in-Tube test measurements performed on the same subjects in three labs excluding those with extremely high TB Response.

			LOA*			
Measure	Comparison	n	Bias* † ± (SDD*)	W-S SD* (95%CI)	ICC (95% CI)	W-S CV% (95% CI)
Nil						
	Lab1 vs. Lab2					
	Unstratified§	91	0.01±(0.11)	±0.04 (0.03, 0.04)	0.89 (0.84, 0.93)	33.63 (29.38, 37.40)
	Concordant Positive#	35	0.00±(0.12)	±0.04 (0.04, 0.06)	0.94 (0.89, 0.97)	35.11 (28.53, 40.63)
	Concordant Negative∧	49	0.01±(0.10)	±0.04 (0.03, 0.05)	0.22 (−0.06, 0.47)	33.56 (27.08, 38.98)
	Lab1 vs. Lab3					
	Unstratified§	91	−0.01±(0.16)	±0.06 (0.05, 0.07)	0.72 (0.61, 0.81)	40.83 (35.50, 45.54)
	Concordant Positive#	35	−0.01±(0.17)	±0.06 (0.05, 0.08)	0.86 (0.74, 0.93)	40.47 (30.89, 48.18)
	Concordant Negative∧	49	−0.02±(0.16)	±0.06 (0.05, 0.07)	0.13 (−0.15, 0.39)	41.91 (34.54, 48.16)
	Lab2 vs. Lab3					
	Unstratified§	91	−0.02±(0.19)	±0.07 (0.06, 0.08)	0.64 (0.50, 0.75)	44.78 (38.78, 50.06)
	Concordant Positive#	35	−0.01±(0.22)	±0.08 (0.06, 0.10)	0.78 (0.61, 0.88)	44.11 (36.00, 50.93)
	Concordant Negative∧	49	−0.02±(0.17)	±0.06 (0.05, 0.08)	−0.14 (−0.40, 0.14)	46.54 (37.31, 54.21)
TB						
	Lab1 vs. Lab2					
	Unstratified§	91	0.19±(2.98)	±1.08 (0.94, 1.26)	0.97 (0.96, 0.98)	28.23 (22.40, 33.05)
	Concordant Positive#	35	0.48±(4.79)	±1.73 (1.40, 2.27)	0.96 (0.92, 0.98)	23.14 (16.98, 27.97)
	Concordant Negative∧	49	0.00±(0.09)	±0.03 (0.03, 0.04)	0.69 (0.51, 0.81)	26.07 (20.30, 30.77)
	Lab1 vs. Lab3					
	Unstratified§	91	0.67±(6.18)	±2.23 (1.95, 2.61)	0.84 (0.77, 0.89)	37.43 (32.06, 42.12)
	Concordant Positive#	35	1.81±(9.62)	±3.47 (2.81, 4.55)	0.76 (0.58, 0.87)	31.32 (25.36, 36.32)
	Concordant Negative∧	49	−0.04±(0.19)	±0.07 (0.06, 0.09)	0.39 (0.13, 0.60)	39.89 (31.87, 46.55)
	Lab2 vs. Lab3					
	Unstratified§	91	0.48±(4.28)	±1.54 (1.35, 1.81)	0.91 (0.87, 0.94)	35.37 (29.77, 40.19)
	Concordant Positive#	35	1.33±(6.61)	±2.38 (1.93, 3.12)	0.85 (0.73, 0.92)	26.99 (19.91, 32.57)
	Concordant Negative∧	49	−0.05±(0.20)	±0.07 (0.06, 0.09)	0.32 (0.05, 0.55)	40.57 (32.31, 47.42)
TB Response						
	Lab1 vs. Lab2					
	Unstratified§	91	0.19±(2.97)	±1.07 (0.94, 1.26)	0.97 (0.96, 0.98)	43.91 (36.95, 49.91)
	Concordant Positive#	35	0.48±(4.77)	±1.72 (1.39, 2.26)	0.96 (0.92, 0.98)	42.04 (33.20, 49.32)
	Concordant Negative∧	49	0.00±(0.08)	±0.03 (0.03, 0.04)	0.70 (0.53–0.82)	42.49 (33.84, 49.66)
	Lab1 vs. Lab3					
	Unstratified§	91	0.68±(6.17)	±2.23 (1.94, 2.61)	0.84 (0.77, 0.89)	55.39 (47.83, 62.03)
	Concordant Positive#	35	1.82±(9.61)	±3.47 (2.80, 4.54)	0.76 (0.58, 0.87)	51.17 (39.96, 60.33)
	Concordant Negative∧	49	−0.03±(0.11)	±0.04 (0.03, 0.05)	0.56 (0.34, 0.72)	57.86 (47.00, 66.98)
	Lab2 vs. Lab3					
	Unstratified§	91	0.50±(4.29)	±1.55 (1.35, 1.81)	0.91 (0.87, 0.94)	57.06 (48.89, 64.20)
	Concordant Positive#	35	1.35±(6.64)	±2.40 (1.94, 3.14)	0.85 (0.73, 0.92)	51.71 (41.14, 60.46)
	Concordant Negative∧	49	−0.03±(0.11)	±0.04 (0.03, 0.05)	0.63 (0.43, 0.77)	61.74 (49.35, 72.03)

* LOA, bias, SDD, and W-S SD are in IU/mL of IFN-γ.

† Directionality for bias and LOA comparisons: Lab1-Lab2, Lab1-Lab3, Lab2-Lab3.

§ Includes 7 subjects with discordant QFT-GIT interpretations.

# Concordant positive among results from all 3 labs.

∧ Concordant negative among results from all 3 labs.

Bias, upper and lower LOA, W-S SD, and their 95% confidence intervals (CIs) for TB Response using an alternative stratification scheme (<0.10 IU/mL, 0.10 to 0.60 IU/mL, and >0.60 IU/mL) based on the subject's mean value from the three labs are shown in Table 6. These results indicate a similar trend of increasing variability with increasing TB Response. The values for the middle group (0.10 IU/mL to 0.60 IU/mL), are intended to provide an estimate of the variability of TB Response surrounding the assay cutoff. W-S SD for this group ranged from ±0.08 IU/mL to ±0.16 IU/mL with the largest upper 95% CI boundary for this group being 0.25 IU/mL (Lab1 vs. Lab 2).

**Table 6:** W-S SD, bias, and LOA in three strata based on the mean TB Response for Lab1, Lab2, and Lab3.

Comparison	n	Bias (95% CI)	Lower LOA (95% CI)	Upper LOA (95% CI)	W-S SD (95% CI)
Lab1 vs. Lab2					
mean* >0.60	34	0.50 (−0.37, 1.36)	−4.34 (−5.84, −2.85)	5.33 (3.84, 6.83)	±1.75 (1.41, 2.30)
mean* 0.10 to 0.60	14	0.00 (−0.13, 0.12)	−0.44 (−0.66, −0.22)	0.43 (0.21, 0.65)	±0.16 (0.11, 0.25)
mean* <0.10	43	0.00 (−0.01, 0.01)	−0.07 (−0.09, −0.05)	0.08 (0.06, 0.10)	±0.03 (0.02, 0.04)
Lab1 vs. Lab3					
mean* >0.60	34	1.88 (0.15, 3.61)	−7.84 (−10.84, −4.85)	11.61 (8.61, 14.61)	±3.51 (2.83, 4.62)
mean* 0.10 to 0.60	14	−0.08 (−0.19, 0.03)	−0.46 (−0.65, −0.26)	0.30 (0.10, 0.49)	±0.14 (0.10, 0.22)
mean* <0.10	43	−0.02 (−0.03, 0.00)	−0.10 (−0.13, −0.08)	0.07 (0.04, 0.09)	±0.03 (0.03, 0.04)
Lab2 vs. Lab3					
mean* >0.60	34	1.39 (0.19, 2.58)	−5.34 (−7.41, −3.26)	8.11 (6.04, 10.18)	±2.43 (1.96, 3.19)
mean* 0.10 to 0.60	14	−0.07 (−0.14, −0.01)	−0.29 (−0.40, −0.18)	0.14 (0.03, 0.25)	±0.08 (0.06, 0.13)
mean* <0.10	43	−0.02 (−0.03, −0.01)	−0.11 (−0.13, −0.08)	0.07 (0.04, 0.09)	±0.03 (0.03, 0.04)

95% CI = 95% confidence interval; LOA = limit of agreement; W-S SD = within-subject standard deviation.

* Stratifications based on mean TB Response among all three labs.

### Comparison of Results Using Original and Reconciled Data

Correction of the manual data entry errors for 9 subjects changed the test interpretations for six subjects: from positive to negative for three and from negative to positive for three (Table S3). Table S1 shows that correcting manual data entry errors resolved the discordance observed in the original results for five subjects, but generated discordance for another subject. While 11.3% of subjects had discordant interpretations among the three labs using original data, 7.7% had discordant interpretations using reconciled data (Table 2). As shown in Table 3, of the Lab3 comparisons, those involving the original data showed lower agreement than those involving reconciled data, while minimal change was observed for Lab1 vs. Lab2, with lowering of the denominator from 97 to 91. Removal of the six subjects with extremely high Lab3 TB and TB Response values did not change the number of subjects with discordant interpretations because these six subjects were concordantly positive. While 36.4% of subjects with discordance using original data had all TB Responses within 0.25 IU/mL of the cutoff, 85.5% of those with discordance using reconciled data had all TB Responses within 0.25 IU/mL of the cutoff.

Quantitative indices of test variability were lowered by correcting the data entry errors. Comparison of quantitative results of original and reconciled data showed that Lab3 median and mean TB and TB Response values decreased following correction of the misplaced decimal point and exclusion of the six subjects with exaggerated TB and TB Response values (Table 4). Median and mean TB and TB Response values for Lab1 and Lab2 also decreased with exclusion of these six subjects. Quantitative variability in TB and TB Response values decreased with data reconciliation as demonstrated by reductions in LOA, W-S SD, ICC, and W-S CV% when unstratified results from each pair of labs were compared using original data (Table S2) versus reconciled data (Table 5).

## Discussion

We observed substantial within-subject interlaboratory variability in QFT-GIT interpretations and IFN-γ measurements when blood samples collected from the same person at the same time were tested in three different labs. Of the 97 subjects tested in three labs, 11% had discordant QFT-GIT interpretations based on the original reported data. Electronic transfer of data was not possible for one of the three labs testing specimens for this study, and a portion of the variability in test interpretation was associated with manual data entry errors. Data entry errors included data misalignments and a misplaced decimal point that were encountered with manual data entry but not electronic data transfers. All three labs used an automated ELISA workstation to assist in performing QFT-GIT, and this may have avoided additional data entry errors. As compared to manually performed ELISAs, automated ELISA workstations can read specimen barcodes that discriminate subjects and QFT-GIT tube type (i.e., Nil tube, TB Antigen tube, Mitogen tube) and assign OD values to specific specimens. This avoids some inaccuracies that have been attributed in prior studies to data entry errors and transposition of IFN-γ measurements [26].

A third type of error was recognized for six subjects who had exaggerated TB values in one lab due to errors in interpreting OD values when they were over the working range of the ELISA workstation. Certain lots of ELISA kits with higher activity as evidenced by higher OD values for standards tended to have higher ODs for plasma samples and have more TB ODs above the working range for the ELISA readers (data not shown). Data from the six subjects with OD values over the working range were excluded from the reconciled dataset. Removal of these subjects with methodological errors did not appreciably alter interpretation agreement because all were concordantly positive.

Corrections of data entry errors made a substantial difference in interpretative agreement between each lab and among all three labs. When reconciled data from Lab1 vs. Lab2, Lab1 vs. Lab3, or Lab2 vs. Lab3 were compared, 94.5%, 93.4%, and 96.7% of interpretations agreed, respectively. However, among all three labs, 92.3% of subjects had concordant results after the data were reconciled.

Several pieces of evidence suggest that the majority of discordance in QFT-GIT interpretation remaining after data reconciliation was due to variability in measuring TB Response. While none of the subjects with discordance attributed to data entry errors had all TB Response values within 0.25 IU/mL of the cutoff separating positive and negative interpretations, 86% of those with discordance after data were reconciled had all TB Response values within this range. Additionally, 37% of the subjects who had one or more TB Response values within this range after data were reconciled had discordance, but none of the subjects without a TB response within this range had discordance. These statistics do not describe the actual magnitude of variability in TB Response.

We examined the magnitude of variability in TB Response and the two IFN-γ measurements used to calculate TB Response. Of the many indices of variability, LOA may be the most informative. LOA is expressed in units of test measurement and includes bias. W-S CV% masks the impact of IFN-γ concentration magnitude on variability, while ICC and W-S SD do not take into account the bias between measurements. Variability, as measured by LOA, was greater for higher IFN-γ measurements. This was observed for Nil, TB, and TB Response, but because TB and TB Response values tended to be larger than Nil values, greater variability was observed in TB and TB Response, especially for subjects with concordant positive interpretations. Because TB Response is calculated from two measurements, its variability could be greater than the variability in measurements used in the calculation (i.e., TB and Nil). Additionally, because Nil and TB are measured in the same ELISA, subtraction of Nil from TB could reduce variability in TB Response by compensating for interassay bias if the bias was constant regardless of the level of IFN-γ measured. However, we observed that (1) the bias in measuring IFN-γ concentration was not constant, (2) the variability in TB Response tracked the variability in TB, and (3) subtracting Nil did not fully compensate for variability in TB when calculating TB Response. Another reason for lower quantitative variability for people with negative results is that the TB Response is constrained to a relatively small range (typically <0.35 IU/mL) compared to the TB Response for those with positive results.

While subjects with concordant positive interpretations had more variability in TB Response than those with concordant negative interpretations, the variability near the cutoff is of greater importance because of its effect on interpretive agreement. Bland-Altman analysis allows assessment of variability in paired measurements and identifies the range of measurements encompassing 95% of TB Response variability associated with repeat testing. Because variability is not uniform across the range of TB Response values, applying a global measure of variability derived from the entire range may not be suitable near the cutoff. Among the 14 subjects with a mean TB Response of 0.10 through 0.60 IU/mL (i.e., 0.35±0.25 IU/mL), which included 6 of the 7 subjects with discordant QFT-GIT interpretations, the upper LOA was as high as 0.43 IU/mL and the lower LOA was as low as −0.46 IU/mL (Table 6). The 95% CIs for LOAs may be relatively large because of the small number of subjects with mean TB Response values near the cutoff. Clinicians, naive to the direction of comparison, can expect results from a second lab to be within 0.46 IU/mL of the first with 95% certainty. Because this estimate of variability is determined for a range (i.e., 0.10 through 0.60 IU/mL), it overestimates variability for TB Response values near 0.10 IU/mL and underestimates variability for TB Response values near 0.60 IU/mL. Another consideration is that for a particular TB Response, changes in only one direction can alter test interpretation.

The amount of uncertainty in interpreting QFT-GIT that is acceptable has not been established. Whereas LOA encompasses a range for 95% of the test-retest differences, bias ± W-S SD encompasses 52% of the variability expected with retesting [30]. W-S SD also reflects the variability relative to the true value such that 68% of measurements will be within one W-S SD of the theoretical true value (typically estimated as the subject's mean value) [30]. W-S SD for TB Response was as high as 0.16 IU/mL for subjects with mean TB Response near the cutoff (i.e., 0.10 through 0.60 IU/mL). W-S SD, which is also referred to as “wobble”, is intended to describe random variation. What we measured as interlaboratory bias could be misinterpreted as random variation if testing were performed in a random selection of laboratories.

We harmonized testing methods as much as possible, so that there were no differences in delays to incubation, incubation time, incubation temperature, and minimal differences in duration of storage. However, there were areas where consistency could not be maintained. For example, labs used QFT-GIT kits with different lot numbers, different automated ELISA workstations, different calibration curves, and different reporting methods. Greater variability may have occurred with less harmonization of test methods.

Various borderline zones around the cutoff have been proposed to address variability [14], [15], [18]–[20], [34]. However, prior investigations have not considered interlaboratory variability or the impact of non-uniform variability in measuring TB Response. Most prior investigations of variability have been challenged to analyze relatively small sample sizes. The small number of subjects near the cutoff also challenged our stratified analysis. Despite the lack of available data from interlaboratory reproducibility studies, our estimates of discordance (11.3% to 7.7%) seem to be in keeping with those seen in intralaboratory between-run estimates of discordance [13], [18]–[20].

Interlaboratory variability is a symptom of a larger problem of IGRA imprecision. IGRA imprecision may also explain a portion of the variability encountered with serially performed IGRAs among healthcare workers [10]–[24]. We measured test variation that is not attributable to subject variation (e.g., due to new infection, treatment, or fluctuations in immune status). Blood samples were collected at the same time to exclude the effect of subject variation due to time. Additional studies are needed to assess IGRA imprecision and understand the components of variation seen in serial testing. The imprecision demonstrated with serial testing and by interlaboratory variability is also relevant when interpreting individual or initial IGRA results.

In conclusion, greater interlaboratory variability was associated with manual data entry and higher IFN-γ measurements. Manual data entry should be avoided. Our data suggest that variability in measuring TB Response may affect QFT-GIT interpretation, especially when near the cutoff. Therefore, consideration should be given to interpreting such responses as “borderline” rather than negative or positive, and clinical decisions regarding treatment or the need to repeat these tests should be based on individualized clinical judgment considering the risk of infection, the risk of disease, and the proximity of the TB Response to the cutoff. In the population we studied, interpreting TB Response values of 0.10 through 0.60 as “borderline” would have avoided most changes in test interpretation due to measurement variability. However, this may not be the appropriate range for the entire population for whom QFT-IT is recommended. Additional studies are needed to determine the optimal range of values for borderline results and to explore the impact of using a borderline interpretation.

## Supporting Information

Table S1 QuantiFERON-TB Gold In-Tube test results for subjects with discordant interpretations.
(DOC)

Table S2 Quantitative indices of variability using original data.
(DOC)

Table S3 QuantiFERON-TB Gold In-Tube test results before and after correction of data entry errors.
(DOC)

Table S4 TB and TB Response Values for 7 subjects with highest Lab3 Values.
(DOC)
