# Supplementary material for: Within-Subject Interlaboratory Variability of QuantiFERON-TB Gold In-Tube Tests
Source: PLoS One. 2012 Sep 6;7(9):e43790. doi: 10.1371/journal.pone.0043790 (PMC3435391; doi:10.1371/journal.pone.0043790)
Supplement: Table S1 — QuantiFERON-TB Gold In-Tube test results for subjects with discordant interpretations. (DOC) [file pone.0043790.s001.doc]

**Table S1.** QuantiFERON-TB Gold In-Tube test results for subjects with discordant interpretations.

|  | **Lab1** | | | **Lab2** | | | **Lab3 (original)** | | | **Lab3 (corrected)** | | |
| --- | --- | --- | --- | --- | --- | --- | --- | --- | --- | --- | --- | --- |
| **Subject ID** | **Nil** | **TB Response** | **Interp.^a^** | **Nil** | **TB Response** | **Interp.^a^** | **Nil** | **TB Response** | **Interp.^a^** | **Nil** | **TB Response** | **Interp.^a, b^** |
| **Discordant with original data only** | | | |  |  |  |  |  |  |  |  |  |
| 74 | 0.068 | -0.004 | Negative | 0.042 | 0.004 | Negative | 0.139 | 46.414 | Positive | 0.043 | 0.002 | **Negative** |
| 75 | 0.072 | 0.007 | Negative | 0.078 | 0.019 | Negative | 0.036 | 0.388 | Positive | 0.060 | 0.033 | **Negative** |
| 76 | 0.076 | 0.019 | Negative | 0.056 | 0.006 | Negative | 0.062 | 2.928 | Positive | 0.065 | 0.007 | **Negative** |
| 77^c^ | 0.119 | 12.234 | Positive | 0.101 | 10.857 | Positive | 0.185 | 0.123 | Negative | 0.139 | 46.414 | **Positive** |
| 80^c^ | 0.309 | 8.302 | Positive | 0.415 | 10.041 | Positive | 0.043 | 0.002 | Negative | 0.393 | 46.160 | **Positive** |
| **Discordant with reconciled data only** | | | |  |  |  |  |  |  |  |  |  |
| 79 | 0.063 | 0.317 | Negative | 0.049 | 0.190 | Negative | 0.065 | 0.007 | Negative | 0.036 | 0.388 | **Positive** |
| **Discordant with both original and reconciled data** | | | | | |  |  |  |  |  |  |  |
| 45 | 0.066 | 0.495 | Positive | 0.060 | 0.299 | Negative | 0.051 | 0.489 | Positive | 0.051 | 0.489 | Positive |
| 47 | 0.066 | 0.404 | Positive | 0.046 | 0.287 | Negative | 0.062 | 0.337 | Negative | 0.062 | 0.337 | Negative |
| 58 | 0.146 | 0.591 | Positive | 0.112 | 0.003 | Negative | 0.086 | 0.207 | Negative | 0.086 | 0.207 | Negative |
| 70 | 0.089 | 0.373 | Positive | 0.059 | 0.465 | Positive | 0.072 | 0.314 | Negative | 0.072 | 0.314 | Negative |
| 87 | 0.096 | 0.176 | Negative | 0.070 | 0.435 | Positive | 0.254 | 0.473 | Positive | 0.254 | 0.473 | Positive |
| 90 | 0.031 | 0.125 | Negative | 0.059 | 0.434 | Positive | 0.047 | 9.952 | Positive | 0.047 | 0.558 | Positive |

Nil concentrations, TB Responses, and QFT-GIT interpretations are shown for the 12 subjects with discordant QFT-GIT interpretations using original data (11 subjects) or reconciled data (7 subjects). Correcting entry errors resolved the discordance observed in the original results for 5 subjects (#74, #75, #76, #77, and #80) but generated discordance for 1 other subject (#79). Two subjects (#77 and #80) were ultimately removed from the reconciled dataset because of extremely high TB and TB Response values and inability to verify such high OD values.

Nil = IFN-γ concentrations (IU/mL) in plasma from the Nil tube of the QuantiFERON-TB Gold In-Tube test (QFT-GIT); TB = IFN-γ concentrations (IU/mL) in plasma from the TB tube of QFT-GIT; TB Response = TB minus Nil; Interp. = Interpretation.

^a^ Underlined text indicates the subject’s discordant interpretation.

^b^ Bold text indicates interpretations that changed when data entry errors were corrected.

^c^ Subject removed from reconciled dataset due to extremely high TB and TB Response values and inability to verify high OD values.
