# Supplementary material for: Within-Subject Interlaboratory Variability of QuantiFERON-TB Gold In-Tube Tests
Source: PLoS One. 2012 Sep 6;7(9):e43790. doi: 10.1371/journal.pone.0043790 (PMC3435391; doi:10.1371/journal.pone.0043790)
Supplement: Table S2 — Quantitative indices of variability using original data. (DOC) [file pone.0043790.s002.doc]

**Table S2.** Quantitative indices of variability using original data.

|  |  |  | **LOA** |  |  |  |
| --- | --- | --- | --- | --- | --- | --- |
| **Measure** | **Comparison** | **n** | **Bias^ +(SDD)** | **W-S SD (95%CI)** | **ICC (95% CI)** | **W-S CV% (95% CI)** |
| Nil |  |  |  |  |  |  |
|  | Lab1 vs. Lab2 | 97 | 0.005 ±(0.11) | ±0.04 (0.03-0.05) | 0.89 (0.85-0.93) | 33.94 (29.74-37.67) |
|  | Lab1 vs. Lab3 | 97 | -0.02 ±(0.19) | ±0.07 (0.06-0.08) | 0.62 (0.48-0.73) | 44.63 (38.62-49.92) |
|  | Lab2 vs. Lab3 | 97 | -0.02 ±(0.21) | ±0.08 (0.07-0.09) | 0.56 (0.41-0.68) | 48.32 (42.11-53.81) |
| TB |  |  |  |  |  |  |
|  | Lab1 vs. Lab2 | 97 | 0.23 ±(3.79) | ±1.37 (1.20-1.59) | 0.96 (0.94-0.97) | 27.69 (22.10-32.33) |
|  | Lab1 vs. Lab3 | 97 | -1.81 ±(23.59) | ±8.51 (7.46-9.91) | 0.48 (0.31-0.62) | 53.02 (43.49-61.08) |
|  | Lab2 vs. Lab3 | 97 | -2.04 ±(23.71) | ±8.55 (7.50-9.96) | 0.45 (0.28-0.59) | 50.93 (41.18-59.09) |
| TB Response |  |  |  |  |  |  |
|  | Lab1 vs. Lab2 | 97 | 0.22 ±(3.76) | ±1.36 (1.19-1.58) | 0.97 (0.96-0.98) | 43.80 (37.05-49.64) |
|  | Lab1 vs. Lab3 | 97 | -1.79 ±(23.54) | ±8.49 (7.44-9.89) | 0.48 (0.31-0.62) | 69.30 (58.17-78.89) |
|  | Lab2 vs. Lab3 | 97 | -2.02 ±(23.66) | ±8.54 (7.48-9.94) | 0.45 (0.28-0.59) | 70.20 (58.89-79.92) |

W-S SD, bias, and LOA are in IU/mL IFN-γ. W-S SD, bias, and LOA are in IU/mL of IFN-γ.

^ Directionality for bias and LOA comparisons: Lab1-Lab2, Lab1-Lab3, Lab2-Lab3.
