# Supplementary material for: Within-Subject Interlaboratory Variability of QuantiFERON-TB Gold In-Tube Tests
Source: PLoS One. 2012 Sep 6;7(9):e43790. doi: 10.1371/journal.pone.0043790 (PMC3435391; doi:10.1371/journal.pone.0043790)
Supplement: Table S3 — QuantiFERON-TB Gold In-Tube test results before and after correction of data entry errors. (DOC) [file pone.0043790.s003.doc]

**Table S3.** QuantiFERON-TB Gold In-Tube test results before and after correction of data entry errors**.**

|  | **TB** | | **Nil** | | **TB Response** | | **Interpretation^a^** | |
| --- | --- | --- | --- | --- | --- | --- | --- | --- |
| **Subject ID** | **Original** | **Corrected** | **Original** | **Corrected** | **Original** | **Corrected** | **Original** | **Corrected** |
| 72 | 46.553 | 2.990 | 0.393 | 0.062 | 46.160 | 2.928 | Positive | Positive |
| 74 | 46.553 | 0.045 | 0.139 | 0.043 | 46.414 | 0.002 | Positive | **Negative** |
| 75 | 0.424 | 0.093 | 0.036 | 0.060 | 0.388 | 0.033 | Positive | **Negative** |
| 76 | 2.990 | 0.072 | 0.062 | 0.065 | 2.928 | 0.007 | Positive | **Negative** |
| 77 | 0.308 | 46.553 | 0.185 | 0.139 | 0.123 | 46.414 | Negative | **Positive** |
| 78 | 0.093 | 0.308 | 0.060 | 0.185 | 0.033 | 0.123 | Negative | Negative |
| 79 | 0.072 | 0.424 | 0.065 | 0.036 | 0.007 | 0.388 | Negative | **Positive** |
| 80 | 0.045 | 46.553 | 0.043 | 0.393 | 0.002 | 46.160 | Negative | **Positive** |
| 90 | 9.999 | 0.605 | 0.047 | 0.047 | 9.952 | 0.558 | Positive | Positive |

Original and corrected TB and Nil IFN-γ concentrations, TB Responses, and interpretations are shown for nine QuantiFERON-TB Gold In-Tube tests with Lab3 data entry errors. Misalignment affected TB, Nil, and TB Response values of eight subjects. Correcting the alignment changed test interpretations for six subjects (from positive to negative for subjects #74, #75, and #76 and from negative to positive for subjects #77, #79, and #80). A misplaced decimal point caused inaccuracy in the reported TB and TB Response values for one subject (# 90) but correction did not alter the interpretation.

^a^ Bold underlined text indicates interpretations that changed when date entry errors were corrected.

Nil = IFN-γ concentrations (IU/mL) in plasma from the Nil tube of the QuantiFERON-TB Gold In-Tube test (QFT-GIT); TB = IFN-γ concentrations (IU/mL) in plasma from the TB tube of QFT-GIT; TB Response = TB minus Nil.
