# Supplementary material for: Within-Subject Interlaboratory Variability of QuantiFERON-TB Gold In-Tube Tests
Source: PLoS One. 2012 Sep 6;7(9):e43790. doi: 10.1371/journal.pone.0043790 (PMC3435391; doi:10.1371/journal.pone.0043790)
Supplement: Table S4 — TB and TB Response Values for 7 subjects with highest Lab3 Values. (DOC) [file pone.0043790.s004.doc]

**Table S4.** TB and TB Response Values for 7 subjects with highest Lab3 Values.

|  |  | **TB** | | | **TB Response** | | |
| --- | --- | --- | --- | --- | --- | --- | --- |
| **Subject ID** |  | **Lab1** | **Lab2** | **Lab3** | **Lab1** | **Lab2** | **Lab3** |
| 81* |  | 18.42 | 11.77 | 102.45 | 18.32 | 11.73 | 102.32 |
| 91* |  | 10.12 | 11.63 | 54.93 | 10.08 | 11.59 | 54.88 |
| 92* |  | 10.12 | 11.63 | 54.93 | 10.07 | 11.48 | 54.58 |
| 77* |  | 12.35 | 10.96 | 46.55 | 12.23 | 10.86 | 46.41 |
| 80* |  | 8.61 | 10.46 | 46.55 | 8.302 | 10.041 | 46.16 |
| 01* |  | 15.00 | 14.60 | 37.43 | 14.91 | 14.49 | 37.35 |
| 52^†^ |  | 16.23 | 17.42 | 18.24 | 16.00 | 17.35 | 18.14 |

* Excluded due to methodological errors that caused exaggerated TB IFN-γ concentrations.

^†^ Next highest Lab3 TB and TB Response values shown for reference and not excluded.
